# An Adaptive Neuromuscular Controller for Assistive Lower-Limb Exoskeletons: A Preliminary Study on Subjects with Spinal Cord Injury

**DOI:** 10.3389/fnbot.2017.00030

**Published:** 2017-06-20

**Authors:** Amy R. Wu, Florin Dzeladini, Tycho J. H. Brug, Federica Tamburella, Nevio L. Tagliamonte, Edwin H. F. van Asseldonk, Herman van der Kooij, Auke J. Ijspeert

**Affiliations:** ^1^Biorobotics Laboratory, École Polytechnique Fédérale de LausanneLausanne, Switzerland; ^2^Department of Biomechanical Engineering, University of TwenteEnschede, Netherlands; ^3^Fondazione Santa Lucia (IRCCS)Rome, Italy; ^4^Department of Biomechanical Engineering, Delft University of TechnologyDelft, Netherlands

**Keywords:** exoskeleton, locomotion, spinal cord injury, neuromuscular controller, biomechanics

## Abstract

Versatility is important for a wearable exoskeleton controller to be responsive to both the user and the environment. These characteristics are especially important for subjects with spinal cord injury (SCI), where active recruitment of their own neuromuscular system could promote motor recovery. Here we demonstrate the capability of a novel, biologically-inspired neuromuscular controller (NMC) which uses dynamical models of lower limb muscles to assist the gait of SCI subjects. Advantages of this controller include robustness, modularity, and adaptability. The controller requires very few inputs (i.e., joint angles, stance, and swing detection), can be decomposed into relevant control modules (e.g., only knee or hip control), and can generate walking at different speeds and terrains in simulation. We performed a preliminary evaluation of this controller on a lower-limb knee and hip robotic gait trainer with seven subjects (*N* = 7, four with complete paraplegia, two incomplete, one healthy) to determine if the NMC could enable normal-like walking. During the experiment, SCI subjects walked with body weight support on a treadmill and could use the handrails. With controller assistance, subjects were able to walk at fast walking speeds for ambulatory SCI subjects—from 0.6 to 1.4 m/s. Measured joint angles and NMC-provided joint torques agreed reasonably well with kinematics and biological joint torques of a healthy subject in shod walking. Some differences were found between the torques, such as the lack of knee flexion near mid-stance, but joint angle trajectories did not seem greatly affected. The NMC also adjusted its torque output to provide more joint work at faster speeds and thus greater joint angles and step length. We also found that the optimal speed-step length curve observed in healthy humans emerged for most of the subjects, albeit with relatively longer step length at faster speeds. Therefore, with very few sensors and no predefined settings for multiple walking speeds or adjustments for subjects of differing anthropometry and walking ability, NMC enabled SCI subjects to walk at several speeds, including near healthy speeds, in a healthy-like manner. These preliminary results are promising for future implementation of neuromuscular controllers on wearable prototypes for real-world walking conditions.

## 1. Introduction

The challenges of developing an effective controller for assistive and rehabilitative robotic devices stem from both incomplete knowledge of healthy neurophysiology and biomechanics and the difficulty in translating such knowledge, however incomplete, into control algorithms. While observed properties of human gait can be reproduced (e.g., joint trajectories), it is unclear how to produce gait that can adapt to a variety of situations and terrains. For individuals with impaired motor functions, active and natural interaction between user and device is crucial for promoting motor recovery and increasing brain plasticity (Rossignol, 2000; Poon, 2004; Rossini and Dal Forno, 2004). Therefore developing a controller that is safe and intuitive to operate, responsive to the user's intentions, and adapts to any walking situation is an unsolved but necessary challenge.

Controllers of assistive exoskeletons generally uses predefined movement patterns. This may entail imposing a specific walking pattern (e.g., early versions of Lokomat, Colombo et al., 2000), which does not require active user involvement and thus may encourage slacking (Schmidt and Bjork, 1992) and reduce motor recovery capacity. Another class of controllers mitigates this problem by assisting the patient when needed [e.g., when the patient's movement is deviated from the desired pattern (Blaya and Herr, 2004; Sup et al., 2008)]. This can be achieved by modulating the stiffness and damping properties of the controller, and knowing these properties also allows controller actions to be stable and predictable. However, to achieve walking at different speeds, for example, these parameters need to be tuned at particular instances in the gait cycle and for each speed (Sup et al., 2009). Multiple reference trajectories for a variety of speeds and situations are needed to cover a wide range of locomotor behaviors, which may require laborious tuning by experimenters, clinicians, or the users themselves.

An alternative approach for adaptive controllers is to utilize time-invariant phase variables. For speed adaptation, these include tibia angular information (Holgate et al., 2009) and virtual constraints (Quintero et al., 2015). With continuous estimation of gait phase, users can accelerate or decelerate within a stride, instead of on a stride-to-stride basis. In comparison to impedance-based control, fewer parameters are needed, and state switching is not necessary. Nonetheless, these methods require accurate sensing of relevant phase variables, and it is uncertain how these methods can be extended beyond speed adaptation, where bio-inspired approaches may have more potential.

User intention is another challenge, and some simple and unambiguous user interface solutions include manual inputs (e.g., push-button) or voice commands. However, these user-activated gaits may be too generic and therefore susceptible to the lack of interaction discussed previously. They also do not address multi-joint level human-machine interaction. However, requiring the user to command lower levels of control (e.g., actuate multiple degrees of freedom) can also lead to high cognitive demands (Tucker et al., 2015). Hence there is a need to find the right balance among reducing the degrees of freedom to be controlled, effective subject involvement, and adaptability.

One interesting approach to encourage shared control that is amenable to different gait conditions is myoelectric control, which uses electromyographic activity (EMG) to generate command signals. Myoelectric control does not require reference signals, and users can actively command their device with modulation of their own muscle signals (Fleischer et al., 2005; Ferris et al., 2006). However, this method relies on clean and reliable signals from functional muscles, which will often be impractical or even impossible to obtain with paraplegics due to their motor control problems.

To promote positive shared user-machine control, bio-inspired controllers that mimic the userÃćÂĂÂŹs own neuromuscular system are one potential solution. Current approaches include leveraging complex musculoskeletal models with virtual Hill-type muscles (Hill, 1938) activated by reflexes (Geyer and Herr, 2010). This model has no predetermined patterns of movement, and walking emerges from the interaction of body dynamics, reflex loops, and virtual muscles with the environment. Not only can the model recreate human behavior such as joint kinematics, kinetic measures, and muscle activations, but it can also walk at a variety of speeds and is robust against perturbations and environmental disturbances in simulation (Song and Geyer, 2015). Controller versions of these models, called neuromuscular controllers (NMC) or reflex-based controllers, have also been implemented on lower limb prostheses (Eilenberg et al., 2010; Thatte and Geyer, 2016) and on assistive devices (Dzeladini et al., 2016; Garate et al., 2016) with promising results.

We investigated the capabilities of the NMC with a haptic gait trainer worn by subjects with a Spinal Cord Injury (SCI). This is the first known application of this controller on a knee and hip robotic device with SCI subjects. We hypothesize that the NMC's virtual dynamics and few sensory inputs could generate healthy-like gait at several speeds for subjects with a diverse range of walking abilities. With NMC assistance, we anticipate that simulating biological muscle motion could allow active recruitment of the user's own neuromuscular system, possibly for rehabilitation.

## 2. Materials and methods

### 2.1. Neuromuscular controller (NMC)

**Figure 1:**
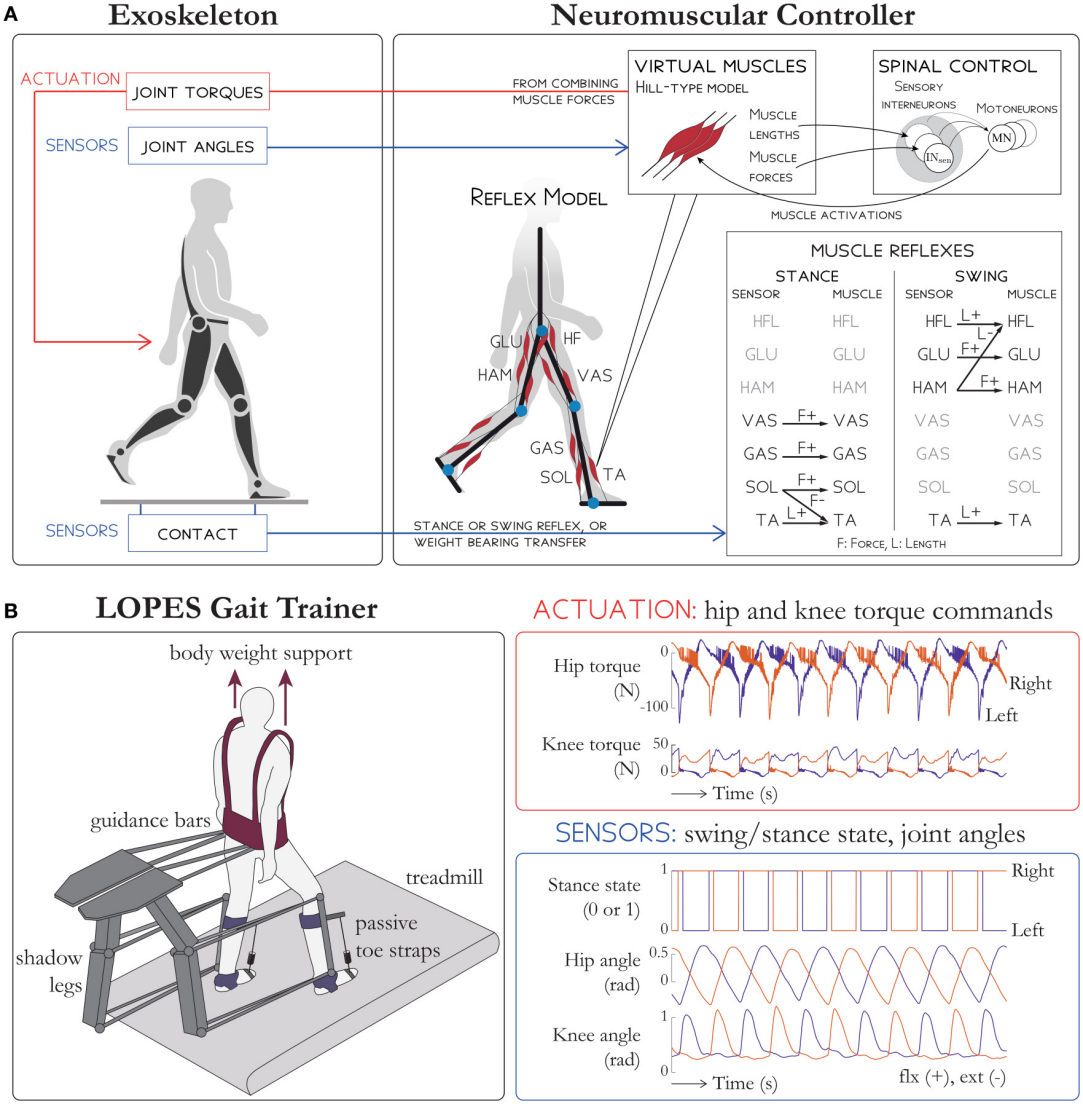
Schematic overview of **(A)** the NMC and **(B)** the LOPES gait trainer. **(A)** Sensors on the exoskeleton are used to detect ground contact. Then, depending on whether the limb is in stance or swing, different reflex rules are activated (shown on the bottom right table). An extra term is added to the hip flexors HFL and hip extensors GLU to facilitate the weight bearing transfer during double support. The reflex loops (which use muscle length, stretching velocity, and tendon force) are then combined to stimulate virtual Hill-type muscles which then generate active torques on exoskeleton joints. **(B)** Subjects in LOPES walk on a treadmill with body weight support. Knee and hip actuation is provided with guidance bars moved by shadow legs, and passive toe straps prevents toe drag. The NMC provides hip and knee torque commands. Sensory input into NMC include stance state, hip angle, and knee angle for both legs. LOPES figure based on Meuleman et al. (2016).

The NMC control paradigm uses a neuromuscular model (NMM) to derive the reference torque pattern used to drive the exoskeleton. The NMM comprises bio-inspired models of muscles, sensors, and neural delays. In this contribution, the NMM used is based on the gait simulation proposed by Geyer and Herr (2010), where the torques applied to the different lower limb joints comprise combined contributions from 14 leg muscles (seven per leg). These virtual muscles are the tibialis anterior, soleus, gastrocnemius, vasti muscles, hamstrings, hip flexors, and glutei muscles (Table 1). The activity of each muscle is the result of different reflex loops that act depending on the gait cycle. During stance, the reflex loops induce higher activity in extensor muscles to favor weight bearing support. When the swing phase is initiated, the reflexes induce a reduction of extensor activity and an increase of flexor activity (see Figure 1A for a detailed description of the NMC).

**Table 1:** Virtual muscles of the neuromuscular controller, their actions, and whether or not they were used on LOPES at the time of testing.

**Muscle**	**Action**	**In LOPES?**
Gluteus (GLU)	Hip extension	Yes
Hip flexor (HFL)	Hip flexion	Yes
Hamstring (HAM)	Hip extension, knee flexion	Yes
Vasti (VAS)	Knee extension	Yes
Gastrocnemius (GAS)	Knee flexion, ankle plantarflexion	No
Soleus (SOL)	Ankle plantarflexion	No
Tibialis anterior (TA)	Ankle dorsiflexion	No

The advantages of this controller over other approaches include robustness, modularity, and adaptability. In particular, the NMC:

- does not require filtering of its inputs (as with myoelectric control),
- can be decomposed into relevant modules (e.g., only knee or hip control), allowing for easy adaptation to different exoskeletons.
- can be modulate the level of assistance (i.e., through scaling commanded torques) to account for subject-specific conditions, such as between legs (to accommodate for left/right asymmetry), between joints of the same leg (to accommodate for joint level asymmetry), and within joints (to accommodate for muscle weakness, i.e., flexor/extension asymmetry).
- can generate walking at different speeds and on different terrains (Song and Geyer, 2012, 2015).

While some of these advantages could be achieved with model-based approaches, the complexity of biological systems hinders traditional models (e.g., impedance-based control) from attaining the same benefits without elaborate control algorithms (as discussed in the Introduction). Likewise, artificial neural networks, such as adaptive frequency oscillators, can also learn to adopt various gait patterns. However, this requires synchronization between the oscillators and the exoskeleton on a step-by-step basis, which arguably takes longer than the quick reactivity of the NMC reflexes.

Instead of constraining a specific motion and resisting against all other external forces, the NMC has the capacity to both work with or against external forces, depending on the direction of the external forces and the current muscle states. For example, during swing at the hip joint, the controller generates a large burst of flexor activity to swing the leg forward. During that period, the NMC's response to an external force on the hip joint would depend on the direction of its application. An extensor torque would act against the controller while flexor torque would act together with the controller. This feature and the ability of the model to produce movement and interaction dynamics in agreement with human locomotion ensures that, barring volitional hindrance by the subject, both the controller and the subject will work in concert. When combined with the modularity aspect of the NMC, this approach allows for easier design of controllers tailored to both the specificity of the subject and of the device.

There are two important differences between the NMM proposed by H. Geyer and the one used here for the NMC. First, to reduce the complexity of the sensors used, a simplified version of the weight transfer reflex, which does not require ground reaction forces (GRFs) but only ground contact information (i.e., the activity of the vastus muscles is decreased or increased depending a filtered version of the ground contact information), was implemented. Second, the trunk balance reflex (proportional-derivative feedback control acting on the trunk to ensure that the trunk stays upright) was not used. This limits the current use of NMC for SCI subjects with very good control of their trunk and thus excludes paraplegics with lesions above C7.

Since the LOPES gait trainer had only knee and hip actuation, we used only the knee and hip NMC modules. This excluded all muscles contributing to the ankle (i.e., the tibialis anterior, soleus, and gastrocnemius). The nominal torques provided by the controller corresponds to those one needed for a human of 80 kg in mass and 1.8 m in height to walk at 1.3 m/s. To produce such a gait, weights on 25 reflex parameters were optimized before the experiment (see Dzeladini et al., 2014, for optimization details). These reflex weights were left unchanged for subject testing. Therefore we did not scale the model to each subject's mass or height (e.g., adjust virtual muscle lengths).

Gains multiplying the nominal torque output of the knee and hip controller were the only parameters used to scale the level of assistance. The gain was applied as a percentage, where 100% was the nominal provided torque and 0% was zero NMC torque, which defaulted to Zero Impedance Mode (ZIM, where the generated torques are to make the device feel as transparent as possible). The assistance gain could also be further tailored to act on specific joints (by multiplying a gain to the knee or hip torque of both legs) or on different legs (by multiplying a gain to the right or left knee and hip torques.)

### 2.2. Experiment

#### 2.2.1. LOPES gait trainer

The haptic gait trainer LOPES (Figure 1B, see Meuleman et al., 2016, for details) consists of shadow legs that help move the subject and a treadmill. The subject is provided body weight support through a harness and is also attached to the device at the waist and at the shank with leg clamps. Active degrees of freedom include shank flexion/extension, thigh flexion/extension and hip abduction/adduction. LOPES can impart up to 70 N-m of knee torque and hip torque (Meuleman et al., 2016), within range of biological torques and therefore able to move the lower limbs of a fully paralyzed subject. The pelvis can also be moved in the forward/aft direction and in the frontal plane. Since the ankle is unactuated, passive toe straps in series with springs were used to prevent toe drag.

#### 2.2.2. Participants

Seven adult subjects walked with a lower limb gait trainer controlled by the NMC. Of the seven subjects, one was healthy (i.e., no neurological deficits, female, 32 years of age, mass *M* 58 kg, height *L* 1.79 m), and the six others had a spinal cord injury (see Table 2 for subject information). Neurological status of SCI patients was assessed using the American Spinal Injury Association (ASIA) and ASIA Impairment Scale (AIS, Kirshblum et al., 2011). Of the SCI subjects (*N* = 6, 2 female, 4 male, 24–52 years of age, mass *M* 69.5 ± 14.9 kg, mean ± s.d., height *H* 1.79 ± 0.07 m), two had incomplete injuries (Group I - AIS level C and D) and the others had a complete injury (Group II - AIS level A). All subjects provided written informed consent prior to the study, according to Institutional Review Board procedures.

**Table 2:** Subject characteristics.

**Subject**	**Group**	**Gender**	**Age (year)**	**Weight (kg)**	**Height (m)**	**Lesion level**	**AIS level**	**Etiology**	**Lesion time (months)**
SHL	–	F	32	58	1.79	–	–	–	–
S1A	I	F	35	48	1.65	T12	C	Trauma	33
S1B	I	M	33	90	1.85	L1	D	Trauma	18
S2A	II	M	52	82	1.78	T7	A	Trauma	13
S2B	II	M	25	64	1.85	T11-T12	A	Trauma	71
S2C	II	M	28	70	1.82	T9	A	Trauma	49
S2D	II	M	24	63	1.80	T7	A	Trauma	61

*SHL is a healthy subject, S1A and S2A are Group I subjects (incomplete injury), and S2A, S2B, S2C, and S2D are Group II subjects (complete injury). Lesion and AIS level from clinical neurological assessment, lesion time is the time from lesion diagnosis to data measurement (in months)*.

#### 2.2.3. Protocol

Subjects walked at a variety of speeds and controller assistance levels, depending on their ability and comfort level. At the beginning of each trial, two experimenters manually maneuvered each leg of the SCI subjects to initiate gait at very slow speeds. Then treadmill speed and controller gains were increased until the subject could walk independently with the controller and without manual assistance. Controller gains and treadmill speed were adjusted based on the subject's needs, level of comfort, and ability and by the clinicians' subjective evaluation of subject safety, gait quality, and perceived exertion. Body weight support (BWS) was also provided, and subjects could use the handrail for support. Trials ranged from ~2 to 5 min long with an average of 112 strides, from which a subset is shown here.

#### 2.2.4. Measurements

We evaluated the joint kinematics and muscle activity as well as the joint torques provided by the controller and virtual muscle properties. Knee and hip joint angles and controller torques were measured from LOPES. The LOPES also measures the total ground reaction force, but not the contribution from each leg. Therefore gait event detection provided to the controller was estimated from the vertical linear velocity of the ankle joint and the angular velocity of the knee, similar to the method reported in O'Connor et al. (2007). We calculated the contribution of handrail usage by subtracting body weight support from the overall bodyweight unloading. Overall unloading was calculated from the average vertical ground reaction force as measured by LOPES and the subject's weight.

We calculated joint power to determine the amount of work performed on the subject by the controller. Joint power (W) was derived from joint angular velocity (time derivative of joint angles) multiplied by joint torques. Joint work (J) was calculated from the integral of positive (or negative) components of power over time over a gait cycle. Virtual muscle lengths, velocities, and activations were determined post-experiment because they were not recorded *in situ*. We simulated the experiment by sending the controller the same sensory inputs (i.e., joint angles, ground contact) as during the experiment.

Electromyographic (EMG) activity of eight muscles was recorded with surface electrodes from the least affected leg (wired Bagnoli system, Delsys, Boston, MA, USA). The muscles measured were the tibialis anterior (TA), soleus (SOL), gastrocnemius medialis (MGAS), vastus lateralis (VL), rectus femoris (RF), biceps femoris (BF), semitendinosus (ST), and gluteus maximus (GMAX). EMG signals were recorded at 1,000 Hz, and the EMG amplifier had a bandwidth of 20–450 Hz. In post-processing, all signals were high-pass filtered with a 20 Hz cutoff frequency (fourth-order Butterworth filter, zero-lag). They were then full-wave rectified and low-pass filtered at 10 Hz (zero-lag) to obtain the linear envelope. Each EMG signal was then normalized by its maximum amplitude over all conditions to obtain a maximum activation of unity.

#### 2.2.5. Analysis

We were primarily interested in whether or not NMC-controlled LOPES could recreate healthy-like gait in SCI subjects. To assess this question, we compared the joint angles and the provided joint torques of NMC walking against joint angles and biological joint torques from healthy shod walking. We also compared EMG patterns of SCI subjects with the healthy subject in LOPES and with the virtual muscle activations of the model to assess changes in muscle activity. As a crude method of evaluating energetic optimality, we investigated the speed-step length relation of SCI subjects with NMC. Finally we further studied two SCI subjects, one with an incomplete lesion to compare walking with ZIM and NMC, and another with a complete lesion to study how changing walking speed affected the NMC.

Since each subject had different levels of walking abilities and impaired behavior (experimental conditions summarized in Table 3), controller settings and treadmill speed varied, making inter-subject comparisons difficult. Therefore only qualitative assessments in magnitudes and trajectories between healthy NMC, SCI NMC, and healthy shod were made for joint angles, torques, and powers at a particular speed and gain. For S1B, S2C, and S2D, this condition was at 0.6 m/s and 100% gain. S2C data was compared at a faster speed (0.7 m/s) because this subject still needed manual assistance at 0.6 m/s. In contrast, S1A and S2A data were compared at a lower speed and gain. S2A was only able to walk with a combination of NMC and manual assistance. We only show data of the same leg from which EMG measurements were made but acknowledge some small asymmetrical behavior could exist.

**Table 3:** Subject experiment settings in NMC-controlled LOPES.

**Subject**	**EMG**	**BWS**	**AS**	**NMC Level**	**Speed**	**Speed Ind**.	**Speed matched**
	**Leg**	**(%BW)**	**(%BW)**	**(%)**	**(m/s)**	**(m/s)**	**(m/s)**
SHL	R	0	0	100	1.0	0	1.0
S1A	L	31	12	40	0.4 (0.15–0.5)	0	0.3
S1B	L	36	38	100	0.6 (0.4–1.4)	0.6	0.6
S2A	R	60	24	30	0.35	None	0.3
S2B	L	24	34	100	0.6 (0.3–0.6)	0.5	0.6
S2C	R	21	48	100	0.7 (0.4–1.0)	0.7	0.6
S2D	R	38	30	100	0.6 (0.4–1.1)	0.6	0.6

**Figure 2:**
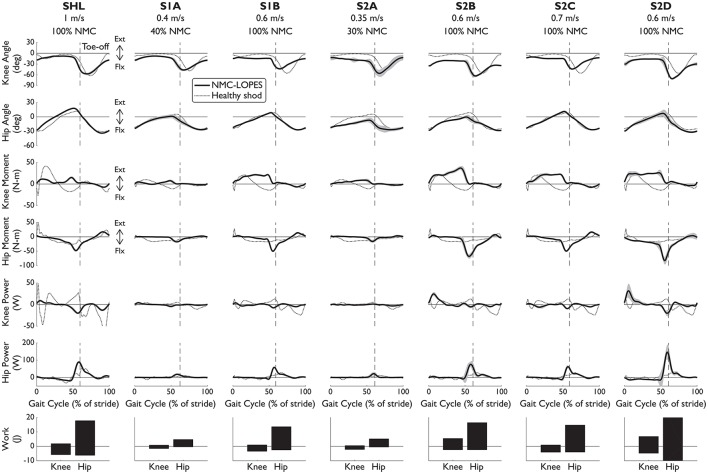
Knee and hip angles and NMC generated moments, power, and work from seven subjects walking with NMC and LOPES. Mean (solid line) and standard deviation (shaded) trajectories are shown along with healthy shod walking (dotted line, see Speed Matched in Table 3) for comparison. Trajectories are shown as a percentage of gait cycle (% of stride) of one leg (corresponds to “EMG leg” in Table 3). Toe-off indicated by dashed vertical line. Ext, positive; Flx, negative.

**Figure 3:**
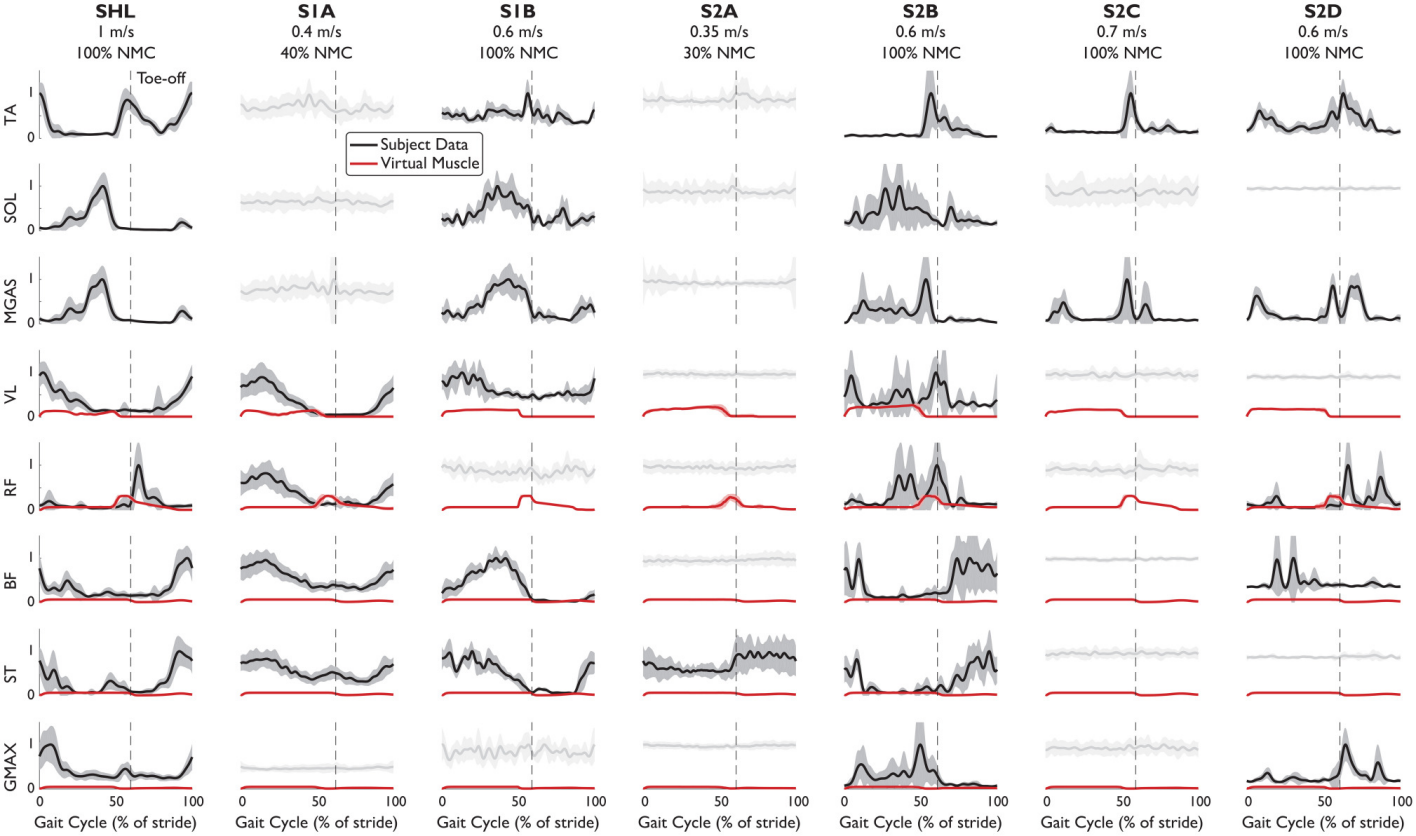
EMG patterns from eight leg muscles of subjects walking with NMC. EMG signals which have met the burst criteria (mean: solid black line), and noisy measurements (light gray) are shown (standard deviation: shaded). Superimposed (red) is muscle activation provided by the virtual muscles in NMC. Trajectories are shown as a percentage of gait cycle (% of stride). Toe-off indicated by dashed vertical line.

**Figure 4:**
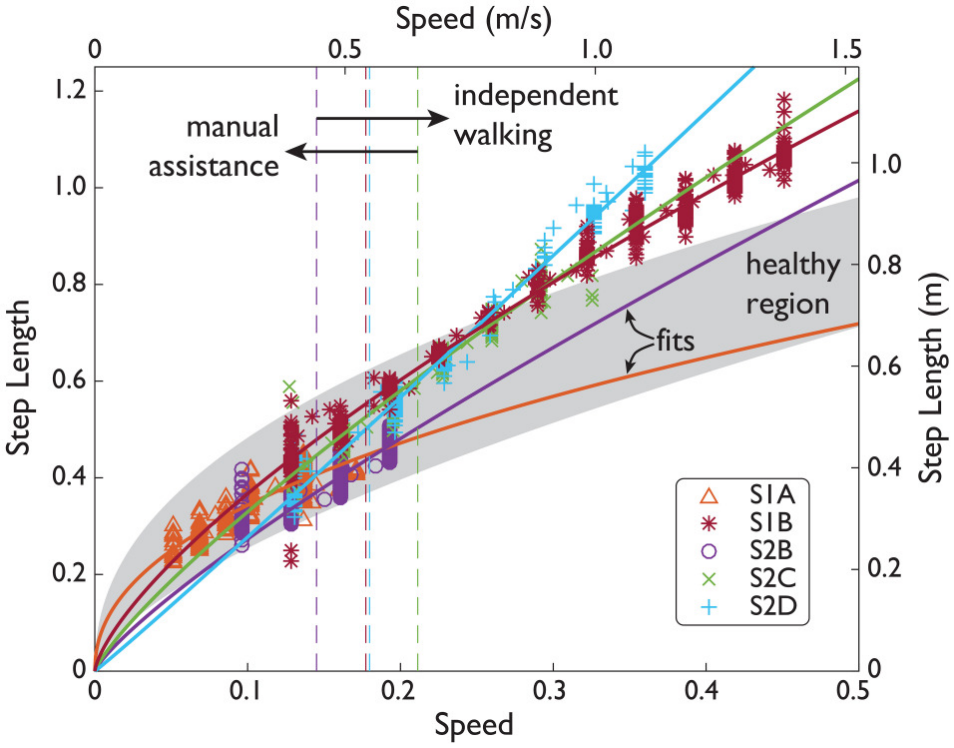
SCI speed and step length over a range of speeds. Subject data (symbols) shown in normalized units (normalized by subject leg length *L*, and gravity *g*) and SI units. Manual assistance threshold (dashed lines) indicates the slowest speed included in subjects' fit (solid lines).

**Figure 5:**
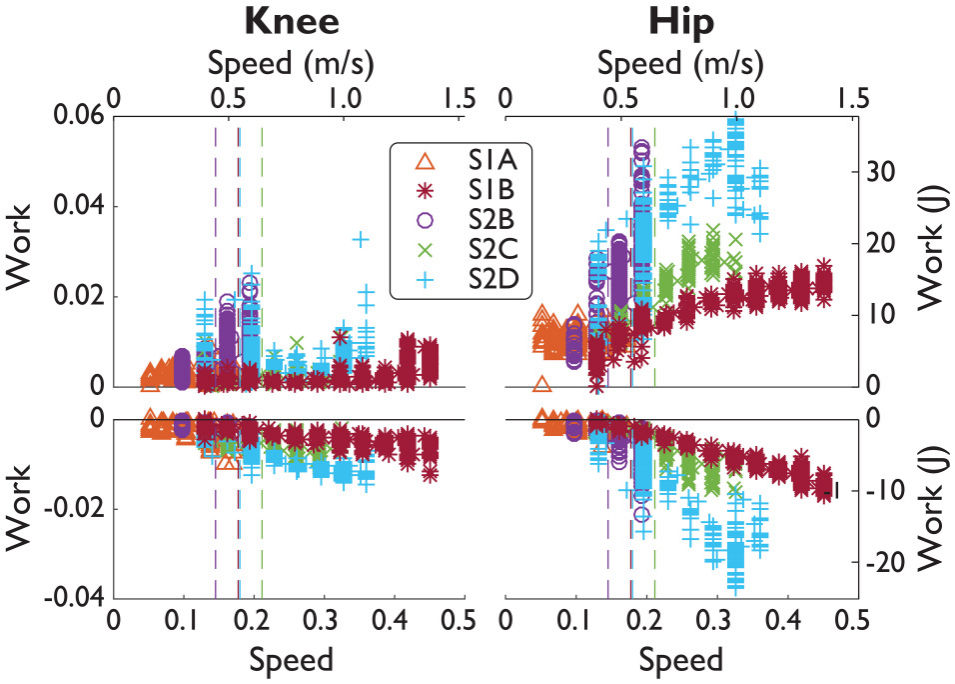
SCI positive and negative joint work over a range of speeds. Subject data (symbols) shown in normalized units (normalized by subject mass *M*, subject leg length *L*, and gravity *g*.) and in SI units. Manual assistance threshold (dashed lines) indicates the slowest speed included in subjects' fit (fits not shown).

*EMG leg indicates from which leg EMG measurements were recorded. BWS is body weight support provided by LOPES as a percentage of body weight. AS is amount of arm support exerted by the subject as a percentage of body weight. Speed is the walking speed exhibited in Figures 2, 3, and the range of speeds for Figures 4, 5 are shown parenthetically. Speed Ind. specifies the speed in which subjects did not need manual assistance. Speed Matched is healthy shod walking speed chosen for comparison against walking with NMC*.

A simple burst detection algorithm was used to determine if EMG patterns contained meaningful or noisy signals. Similar to Di Fabio's method (Di Fabio, 1987), we first calculated a 50 ms baseline of non-activity for each muscle signal. Then we evaluated whether or not there was a consecutive 25 ms window of activity that was greater than the mean plus three times the standard deviation of the baseline activity. If this activity existed, then the signal was deemed a viable measurement. As an indication of activation timing, we also calculated the time of peak muscle activation as a percentage of gait cycle. For the tibialis anterior signal only, we searched for the peak near toe-off.

The average EMG traces were also compared against activation signals of its corresponding virtual muscle. Since the NMC is a simplification of the human musculoskeletal system, the vastus lateralis was compared against the modeled vasti activation, the rectus femoris against the hip flexors, biceps femoris and semitendinosus with the hamstring, and the gluteus maximus against the glutei muscle group. Although some subject EMG signals did not contain any activity, it was conceivable that the virtual muscle activations could compensate for the lack of motor function.

For comparison with NMC gait, healthy shod joint measures were derived from one subject (female, 31 years of age, mass *M* 65 kg, height *H* 1.63 m) walking on a treadmill at 0.3, 0.6, and 1.0 m/s. We derived kinematics and inverse dynamics (Opensim, Stanford, CA, USA) from motion capture measurements (Phoenix Technologies, Visualeyez, Canada) and ground reaction forces from an instrumented dual-belt treadmill (Motekforce Link, Amsterdam, the Netherlands).

We made two quantitative comparisons across multiple subjects and trials to determine the general behavior of the NMC. First, we assessed whether the NMC could reproduce the speed-step length relation found in healthy gait (Grieve, 1968) and second, the relation between speed and joint work (Donelan et al., 2002). The first relation represents energetically optimal changes in step length with speed. Healthy subjects have been found to walk with a step length *s* following the power law *s* = α*v*^β^ with β typically reported to be 0.54 ± 0.10 (Collins and Kuo, 2013). To calculate exponent β (and offset α), we applied a linear regression of log*s* = logα + βlog*v* for each SCI subject for all trials without manual assistance (see Table 3 for speed ranges). Since S2B did not walk without manual assistance, his data was excluded from this analysis.

We also determined the relation between speed *v* and joint work *W*. Past studies (Zelik and Kuo, 2010) have found that total joint work should be proportional to speed *W* = *v*^0.28^. However, it is unclear if the same relation holds for individual joints. Instead we simply performed a linear regression on *W* = α + *vβ* to determine the trend β to understand how NMC torques change with speed.

To further illustrate the effect of NMC speed-related changes, we showed biomechanical measures from S2D walking at 0.8, 0.9, and 1.0 m/s at a constant assistance level of 100%. We also compared the joint trajectories of NMC-controlled gait with the controller inactive (i.e., ZIM) with S1A, who was the only SCI subject to have walked without assistance manually provided by the experimenters.

Analysis was performed on a stride-by-stride basis with each measure calculated as the average over all strides within a condition. All values for comparisons across subjects (i.e., speed, step length, joint work) were analyzed in dimensionless form. We performed linear regression to determine speed-related trends for step length and for joint work. Regression coefficient β was statistically significant if its *P*-value was less than 0.05 (*P* < 0.05). Normalization was performed using base units of body mass *M*, leg length *L*, and gravity *g*. Leg length *L* was calculated as 0.530*H* (Contini, 1972). Step length was normalized by *L*, speed by $\sqrt{gL}$, and work by *MgL*. For reporting purposes, statistical data were converted from dimensionless units to SI units using mean normalization constants of *L* = 0.9508 *m*, $\sqrt{gL}=3.05$ *ms*^−1^, and *MgL* = 629 *J*.

## 3. Results

With NMC-controlled LOPES, the SCI subjects were able to walk at various speeds (from 0.6 to 1.4 m/s), faster than typical for ambulatory SCI patients (e.g., average speed from 0.34 to 0.88 m/s van Hedel and EMSCI Study Group, 2009). In comparison, only one of the SCI subjects could walk unsupported in LOPES (S1A at 0.4 m/s in ZIM). Their joint angle trajectories were similar to healthy humans, but joint torques were not, due to the lack of ankle actuation in the device and thus active control. For SCI subjects, body weight support unloaded 21–60% of their body mass *M* and use of handrails contributed an additional 12–48% *M*.

### 3.1. Joint kinematics and kinetics and comparisons with healthy data

The NMC was successful in producing healthy-like walking patterns. NMC joint angles and torques agreed reasonably well with healthy kinematic data and biologically produced torques (Figure 2, first four rows). Differences were found between NMC-provided torques (for both SCI and healthy) and biological torques produced by healthy subjects, including a lack of knee flexion torque near mid-stance and greater hip moment near toe-off. Hip extension torque at heel-strike was also missing. Despite these discrepancies, joint angle trajectories did not seem greatly affected. The torque differences also translated into differences in joint powers (Figure 2, fifth and sixth row), notably more positive hip power around toe-off. On average, more hip work was delivered than knee work (Figure 2, last row).

The interaction between the subject and the NMC-controlled LOPES influences the NMC-provided torques and thus overall gait behavior. For example, healthy subject SHL required less assistance than the SCI subjects. Therefore despite the faster speed and generally larger range of motion, NMC provided SHL with similar or smaller knee and hip torques than for other subjects with the same gain (S1B, S2B, S2C, and S2D) but walking at slower speeds. We also expected the NMC to provide less torque at small gains and slow speeds. Indeed NMC delivered relatively small torques and therefore work to S1A and S2A, both of whom walked at slow speeds (0.4 and 0.35 m/s respectively) and low gain (40 and 30% respectively).

EMG patterns (Figure 3) indicated that the NMC controller could be inducing rhythmic activation patterns in leg muscles of complete SCI subjects. For three subjects with complete paraplegia, meaningful muscle activity was found at the tibialis anterior and medial gastrocnemius. While these muscles were not modeled in the controller and ankle dorsiflexion/plantarflexion was not an actuated degree of freedom in LOPES, the subsequent walking motion may have activated these muscles, intentionally or not by the subject. Unsurprisingly, systematic muscle activity was found in all muscles measured for the healthy subject. EMG activity was also detected for all measured muscles for S2B (complete injury), and perhaps this stems from this subject's comparatively low lesion level (T11-T12).

From the timing of peak EMG activity, muscle activation patterns were more consistent among subjects for the distal muscles than for the proximal muscles (Table 4). Standard deviation of peak time for the distal muscles (TA, SOL, MGAS) ranged from 3 to 7% of stride but was ~7 times greater for the proximal muscles. In particular, the biceps femoris and semitendinosus muscles of SCI subjects differed the most from healthy subject SHL and from each other. S2B (complete injury), who exhibited activity in all muscles, had muscle activation patterns that were similar to those of SHL for most muscles (except VL and GMAX).

**Table 4:** Time (% of stride) of maximum muscle activation in NMC-controlled LOPES.

**Subject**	**TA**	**SOL**	**MGAS**	**VL**	**RF**	**BF**	**ST**	**GMAX**
SHL	57.5	41.5	41.1	2.40	64.9	96.7	91.7	7.40
S1A	–	–	–	13.9	16.4	16.4	17.1	–
S1B	56.3	36.4	43.6	13.3	–	35.5	19.9	–
S2A	–	–	–	–	–	–	70.1	–
S2B	56.8	36.1	53.5	60.0	60.7	82.1	94.8	49.8
S2C	55.8	–	53.4	–	–	–	–	–
S2D	62.8	–	56.0	–	65.9	30.4	–	64.3

*Maximum activation timing reported as a percentage of gait cycle for mean EMG patterns (see Figure 3)*.

While the virtual muscles could serve to supplement missing biological function, the virtual muscles do not seem to differ greatly among subjects, even between SHL and S2A, who only had activity in one muscle. For all subjects, activation signals provided by the virtual muscles were greater for the vasti muscles and hip flexors (compared with subjects' rectus femoris) but small for the hamstring and glutei. The virtual muscle activities are also generally not similar to SHL's EMG activity. This could be due to the weight transfer simplification, which now produces muscle activities that differs from previously reported in simulation (Geyer and Herr, 2010; Dzeladini et al., 2014).

As walking speed increased (along with increases in NMC gain up to 100% assistance), the NMC produced longer step lengths and more joint work. Subjects walked with step lengths that resembled the power law found empirically in healthy gait but were not as energetically optimal due to relatively longer step lengths at faster speeds (Table 5). Four subjects demonstrated the power law with β = 0.70 ± 0.17 (mean ± s.d., mean *R*^2^ = 0.84), and one other exhibited a more linear trend (β = 1.03, *R*^2^ = 0.98). Unlike the other subjects who exhibited the power law, S1A showed a shallower increase in step length. This is likely related to fitting to the subject's slow range of speeds (up to 0.4 m/s). For the subjects who exhibited the power law, the average step length at 1.6 m/s was ~17% greater than for a healthy human.

**Table 5:** Step length *s* and knee and hip joint work (positive *W*^+^ and negative *W*^−^) fit parameters, goodness-of-it, and statistical significance of trend values.

**Parameter**	**Subject**	**Coefficient β±CI**	**Offset α±CI**	***R*^2^**	***P***
*s*	S1A	0.4591 ± 0.0301	0.9880 ± 0.0688	0.8203	0.0000^*^
	S1B	0.7141 ± 0.0095	1.9007 ± 0.0108	0.9837	0.0000^*^
	S2B	0.8152 ± 0.0710	1.7865 ± 0.1234	0.6657	0.0000^*^
	S2C	0.8160 ± 0.0455	2.1572 ± 0.0625	0.8932	0.0000^*^
	S2D	1.0297 ± 0.0174	2.9703 ± 0.0255	0.9815	0.0000^*^
$W_{\text{knee}}^{+}$	S1A	−0.0021 ± 0.0052	0.0023 ± 0.0006	0.0032	0.4284
	S1B	0.0138 ± 0.0023	−0.0020 ± 0.0008	0.2722	0.0000^*^
	S2B	0.0777 ± 0.0257	−0.0040 ± 0.0046	0.1211	0.0000^*^
	S2C	0.0113 ± 0.0082	−0.0005 ± 0.0021	0.0478	0.0068^*^
	S2D	0.0129 ± 0.0088	0.0034 ± 0.0022	0.0313	0.0043^*^
$W_{\text{knee}}^{-}$	S1A	−0.0185 ± 0.0071	−0.0001 ± 0.0008	0.1165	0.0000^*^
	S1B	−0.0107 ± 0.0015	−0.0010 ± 0.0005	0.3555	0.0000^*^
	S2B	−0.0672 ± 0.0066	0.0090 ± 0.0012	0.6132	0.0000^*^
	S2C	−0.0107 ± 0.0093	−0.0041 ± 0.0024	0.0335	0.0241^*^
	S2D	−0.0328 ± 0.0030	0.0006 ± 0.0007	0.6483	0.0000^*^
$W_{\text{hip}}^{+}$	S1A	0.0204 ± 0.0103	0.0083 ± 0.0012	0.0711	0.0001^*^
	S1B	0.0339 ± 0.0020	0.0082 ± 0.0007	0.7565	0.0000^*^
	S2B	0.3173 ± 0.0483	−0.0288 ± 0.0086	0.3942	0.0000^*^
	S2C	0.0851 ± 0.0127	−0.0038 ± 0.0033	0.5399	0.0000^*^
	S2D	0.1258 ± 0.0140	0.0081 ± 0.0035	0.5495	0.0000^*^
$W_{\text{hip}}^{-}$	S1A	−0.0107 ± 0.0031	−0.0000 ± 0.0004	0.1906	0.0000^*^
	S1B	−0.0434 ± 0.0014	0.0056 ± 0.0005	0.9140	0.0000^*^
	S2B	−0.1088 ± 0.0184	0.0145 ± 0.0033	0.3451	0.0000^*^
	S2C	−0.0767 ± 0.0109	0.0108 ± 0.0028	0.5614	0.0000^*^
	S2D	−0.1383 ± 0.0087	0.0175 ± 0.0022	0.7912	0.0000^*^

*Coefficients β and offsets α from linear regression to s = αv^β^ or W = α + vβ. Significant coefficients are indicated by asterisk if p < 0.05. Coefficients and offsets reported in dimensionless units using base units of body mass M, leg length L, and gravitational acceleration g*.

NMC produced greater joint work in response to increases in treadmill speed, more notably at the hip than at the knee (Figure 5, Table 5). On average, positive work trend was 5.1 times greater for the hip than for the knee, and 2.7 times greater for negative work. For significant trends (*p* < 0.05), we found that positive hip work increased at a rate from 2.86 *Wm*^−1^*s* (S1A) to 63.0 *Wm*^−1^*s* (S2B) with mean goodness of fit *R*^2^ = 0.46. Negative hip work trend ranged from −26.6 *Wm*^−1^*s* (S2D) to −1.50 *Wm*^−1^*s* (S1A) with mean *R*^2^ = 0.56. For the knee, positive work coefficient ranged from 2.44 *Wm*^−1^*s* (S2C) to 15.4 *Wm*^−1^*s* (S2B) with mean *R*^2^ = 0.12, and negative work was from −13.3 *Wm*^−1^*s* (S2B) to −2.30 *Wm*^−1^*s* (S2C) with *R*^2^ = 0.35.

### 3.2. Subject S1A (incomplete injury): 40% NMC gain vs. ZIM

**Figure 6:**
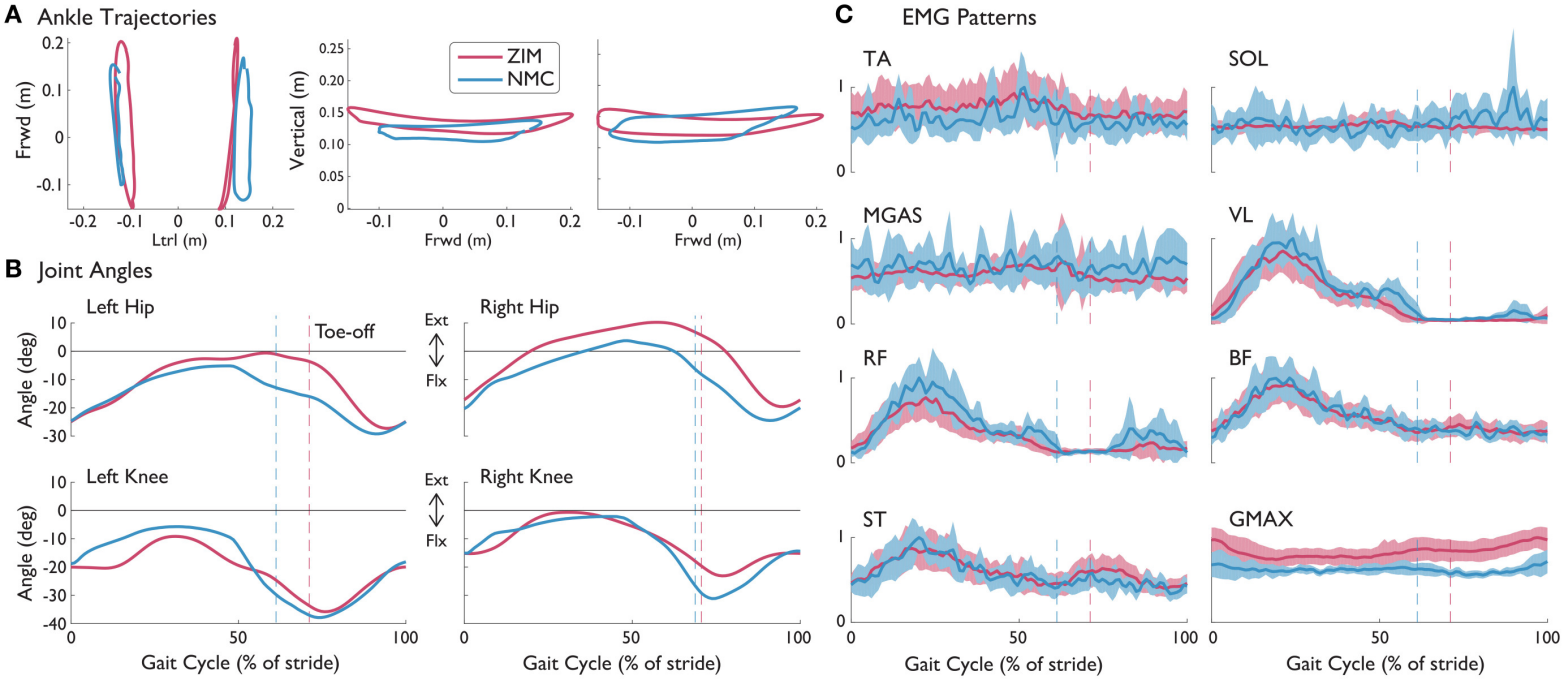
**(A)** Left and right ankle trajectories, **(B)** knee and hip angles, and **(C)** EMG patterns with standard deviations (shaded) from S1A (incomplete SCI injury) with NMC assistance and without (ZIM) at 0.15 m/s. Trajectories are shown as a percentage of gait cycle (% of stride). Toe-off indicated by dashed vertical line. Ext, extension; Flx, flexion.

S1A was the only subject to have walked with LOPES in both ZIM and with the NMC. S1A walked at 0.15 m/s in ZIM and with 40% of NMC assistance. Due to problems in step detection for this subject, only seven strides were analyzed for the NMC condition at this speed while 50 strides were analyzed for zero impedance mode. However, the variability of step parameters are similar for both conditions. NMC served to create shorter strides when compared against zero impedance mode. The average step length with NMC (0.21 ± 0.01 m) was shorter than without (0.28 ± 0.03 m), as demonstrated by ankle trajectories (Figure 6A). In contrast, the average step width with NMC (0.26 ± 0.02 m) was slightly wider than the zero impedance mode (0.23 ± 0.03 m). The NMC created a larger range of motion for the knee but reduced motion for the hip, contributing to shorter step lengths (Figure 6B). EMG activity seemed similar in magnitude and activation pattern, except for the gluteus muscle, where the mean activity was slightly higher on average (Figure 6C).

### 3.3. Subject S2D (complete injury): speed-related changes at 100% NMC gain

We observed that NMC's gait adaptations to different speeds were similar to observations of healthy subjects walking at different speeds. In particular, in response to treadmill speed changes, the NMC automatically modulated the torques exerted on the subject. We evaluated speed-related changes for S2D, who walked at 0.6, 0.9, 1.0 m/s at 100% of NMC assistance. The increase in walking speed produced greater step lengths (0.51 ± 0.02, 0.82 ± 0.02, 0.89 ± 0.02 from slowest to fastest speed) while step width changes showed no trend (0.24 ± 0.03, 0.31 ± 0.02, 0.27 ± 0.02 m).

**Figure 7:**
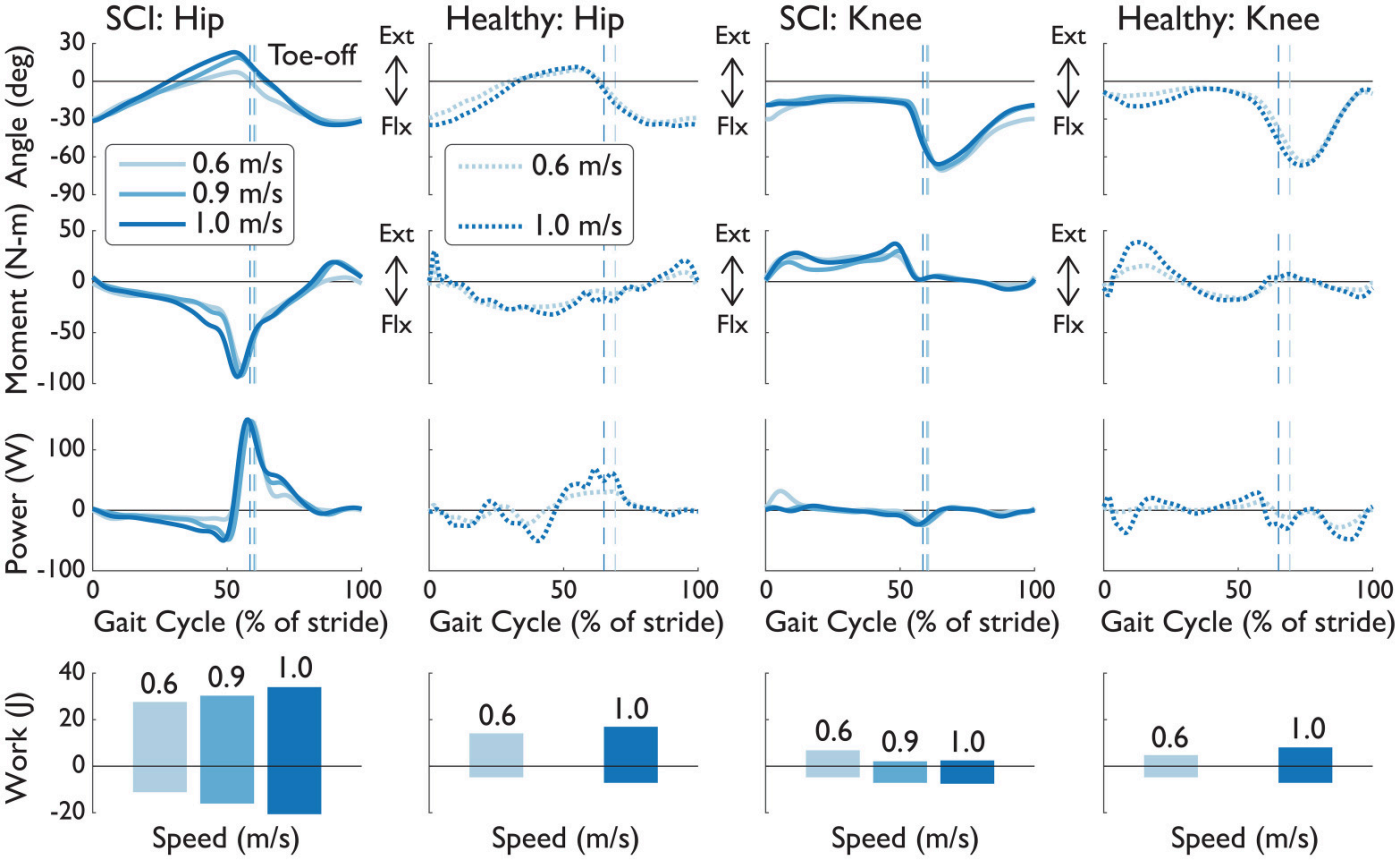
Knee and hip angles, moments, powers, and work from the right leg of S2D (complete SCI injury) while walking with NMC and LOPES. Mean trajectories (solid line) are shown with healthy shod walking (dashed line) at similar walking speeds. Trajectories are shown as a percentage of gait cycle (% of stride). Toe-off indicated by dashed vertical line. Ext, extension; Flx, flexion.

Speed increases led to greater magnitudes in joint angle, similar to healthy humans (Figure 7). In addition, NMC provided more peak torque, especially at the hip, as humans would increase biological torques to walk faster. While little changes were observed in peak powers, positive and negative work did increase with speed (with the exception of positive knee work).

**Figure 8:**
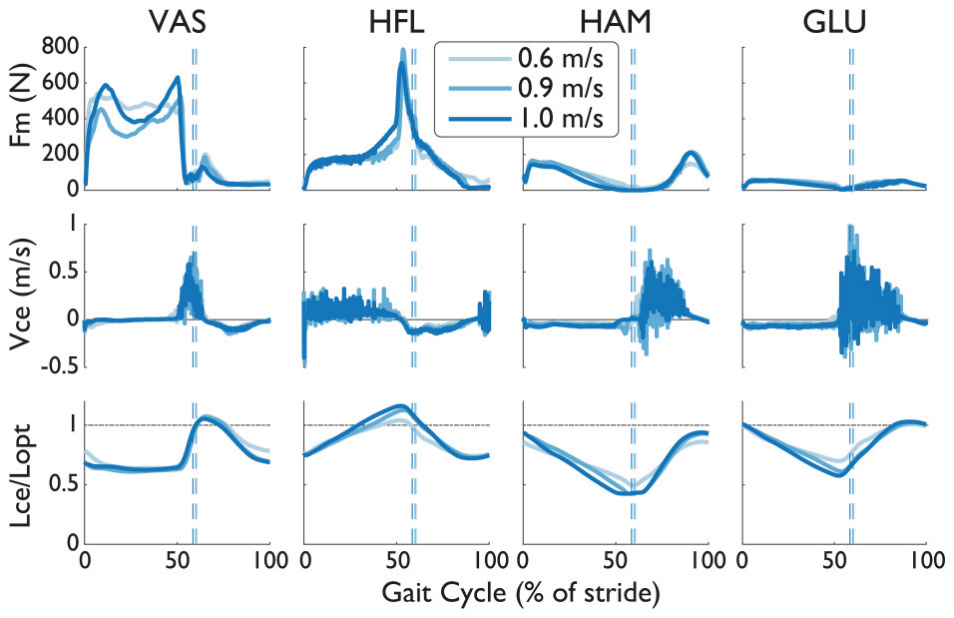
Muscle force (Fm), contraction velocity (Vce), and length (Lce) from four virtual muscles of the NMC with S2D (complete SCI injury) walking in LOPES. The length of the contractile element has been normalized by the optimal length Lopt (dashed-dotted line). Trajectories are shown as a percentage of gait cycle (% of stride). Toe-off indicated by dashed vertical line.

We investigated the muscle force, contractile velocity, and length from NMC's virtual muscles (Figure 8). The speed-related increase in torque was produced mainly by changes in the length of the virtual muscles rather than by velocity. In congruent with how greater speeds induce longer strides, the hip extension muscles (i.e., hamstring and glutei muscles) were more contracted at fast speeds than slow speeds around maximum hip extension (~50% of gait), and the hip flexor muscles were more extended. The vasti muscle did not show much change in length except near heel-strike at the slowest speed. In contrast to the changes in muscle length, there was no visible trend from the contractile velocity of the virtual muscles. The noise-like behavior in these signals are from integration of differential equations in the muscle model (see Dzeladini et al., 2014). Muscle forces also seem to be affected by speed, but peak forces do not seem proportional to speed.

## 4. Discussion

Our preliminary results demonstrated the versatility of the NMC. With very few sensors, SCI subjects were able to walk at multiple speeds, including near healthy speeds, despite the lack of ankle actuation. NMC gait kinematics resembled those of healthy shod walking. With no predefined settings for multiple walking speeds, the NMC also adjusted step length similarly to healthy humans as speed changed. Meaningful EMG activity was also detected in several muscles of SCI subjects, possibly implying functional engagement of the subjects' own muscles.

Several factors could explain the observed differences between NMC-generated torques and biological torques. One source of disparity is the neuromuscular model (NMM), the basis of the controller, generates human-like walking in simulation but cannot fully capture human behavior. For example, compared with biological torques, the model produces a greater hip flexion torque near toe-off, which we also observed with NMC-generated torques. In addition, model parameters from NMM simulation were directly applied to the controller, and therefore user-machine interactions were not taken into account.

**Figure 9:**
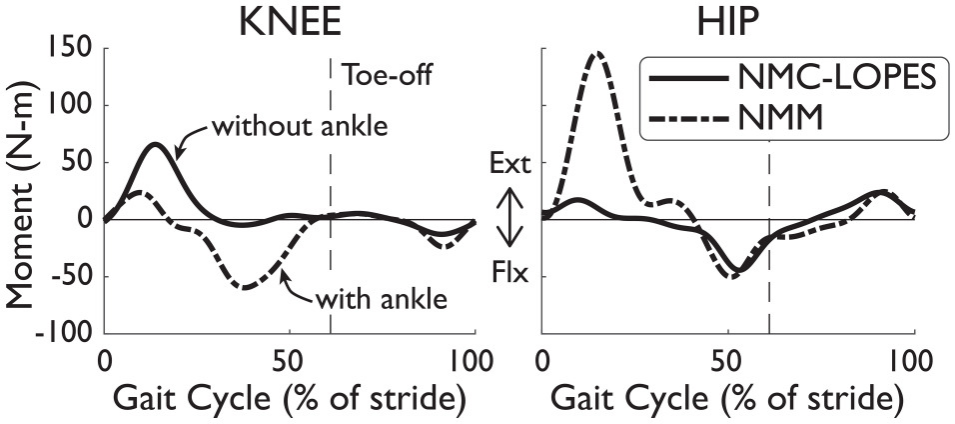
Joint torques differences between the full NMM (Dzeladini et al., 2014) and the NMC-LOPES controller. Without the ankle module, the knee torque from NMC-LOPES (without ankle, solid line) exhibits little flexion near mid-stance in comparison with torques from the full NMC model (with ankle, dashed-dotted line). The large extension hip torque at early stance in the full model is also omitted in the reduced controller. Trajectories are shown as a percentage of gait cycle (% of stride). Toe-off indicated by dashed vertical line.

The lack of ankle actuation is another compelling reason for the differences in NMC and biological torques. For the knee disparities, the virtual biarticular gastrocnemius muscle, which provides knee flexion, was also omitted in the NMC for implementation in LOPES. While the virtual hamstring muscle can also provide knee flexion, nominal behavior of the full NMM (i.e., with ankle, from Dzeladini et al., 2014) is a burst of muscle activity in the gastrocnemius but little in the hamstring during peak knee flexion (Geyer and Herr, 2010). Therefore, without the virtual gastrocnemius muscle, NMC's ability to produce knee flexion torque is reduced. This was also found in simulation by feeding NMM joint angles and footfall patterns into NMC (Figure 9).

The differences in torques at the hip joint is less clear. No virtual muscles were missing at this joint and therefore abnormal behavior was not expected. NMC hip angles were also very similar to healthy angles and is unlikely greatly influencing NMC torques. However, the simplification of the weight bearing algorithm (see Section 2.1), which affects the virtual vasti muscles, glutei muscles, and hip flexors, could be one explanation. The major discrepancies in hip torques occurred during double support (e.g., near early stance and toe-off), which coincides with weight transfer from one leg to the other. In simulation, the missing hip extension torque (Figure 9) of the NMC-LOPES controller at early stance, in comparison to the full NMM, supports this reasoning.

Differences in step length trends between SCI gait and healthy gait with increased speed could be partially explained by body weight support and the use of handrails. These subjects had 21–38% of their body weight unloaded and their use of handrails also provided an additional 12–48% of support. S3D, the subject with the linear trend, had the highest body weight support provided by LOPES. Body weight support has been shown to affect gait kinematics at 75% of BW (van Hedel et al., 2006), and the reported effect on stride length (at greater speeds) was a significant but small increase relative to zero bodyweight support.

Although walking speed was regulated by the treadmill, the NMC is reactive controller that acts only in the sagittal plane and thus gait adjustments could be made by healthy subjects by changing step time or step width. For the SCI subjects, less adjustments were possible due to their impairment, but subjects did employ their arms (as indicated in the previous paragraph) by imparting forces on the handrails. Some arm use may be in response to an unfamiliar device and controller and the fear of tripping. However, some SCI subjects also used their arms to utilize their upper body to assist in propelling their legs forward and in making small lateral corrections. We feel this was unnecessary, as the NMC would swing the leg as soon as it was unloaded, but we did not test this controller on a completely passive subject. We also did not directly quantify or study upper arm effort, but we did ask subjects to decrease their reliance on the handrails if possible.

Pronounced EMG patterns were detected from both incomplete and complete SCI subjects. Some patterns seemed similar to healthy (e.g., TA, BF, and ST of S2B) while others were more aberrant (e.g., MGAS of S2C and S2D). While these patterns may have been induced by the uncontrived NMC gait dynamics, it is difficult to separate in the present study these findings from EMG activity previously found with coordinated stepping movements by physiotherapists (Dietz et al., 1994) and a fixed gait pattern by a driven gait orthosis (Dietz et al., 2002). Nonetheless, as the previous studies have noted, subjects' muscle activities in both the actuated joints of the LOPES and the passive ankle are likely the result of systematic load receptor input during each stride. The implication and veracity of this finding deserves further investigation.

The NMC exhibited speed adaptation by modulating its commanded torques as treadmill speed changed (from 0.4 to 1.4 m/s across subjects), resulting in higher peak torques (see Figure 7) and greater joint work (see Figure 5). These results agree with those from a similar neuromuscular controller for a transtibial prosthesis (Markowitz et al., 2011), which produced greater ankle torque with increased speed (at 0.75, 1.0, and 1.25 m/s). In impedance-based control of a different transtibial prosthesis (Fey et al., 2014), subjects were able to ambulate at ±25% of their comfortable walking speed (from 0.49 to 1.39 m/s across subjects), also with increased ankle torque and power with faster speeds. While these two controllers served to emulate locomotion for missing limbs rather than impaired ones, our controller showed comparable results for speed adaptation. Since few speeds were tested in those studies, it is difficult to assess how well those controllers would reproduce the speed-step length curve (see Figure 4), as we have achieved here.

There were some limitations to this study. We could not compare the NMC against the device's ZIM because SCI subjects were unable to walk without assistance. In addition to testing a small number of subjects, SCI subjects also could not be evaluated at the same speeds and controller settings because each had unique neurological symptoms, and controller gains were manually tuned for their specific walking ability. A different investigation with healthy subjects with the NMC would be appropriate to more fully evaluate the NMC and its ability to lessen the energetic burden of walking (e.g., less metabolic cost). However, as our aim is to restore gait in paraplegic subjects, the controller fared well despite the lack of ankle actuation.

Due to limitations in experimental set up, we also did not evaluate how walking in LOPES affects healthy gait. In particular, we compared NMC to shod walking but did not evaluate how biological joint torques for a healthy subject walking in LOPES (calculated from inverse dynamics) under ZIM would differ from shod walking. In addition, some of the differences between NMC and shod joint angles could be due to dissimilarities between LOPES-measured angle and kinematics from motion capture.

The NMC was not optimized for subject anthropometry to provide subject-specific assistance or at multiple walking speeds. Although subject-NMC interaction allowed for slow walking speeds, the NMC cannot function at speeds slower than 0.6 m/s in simulation. These issues are to be addressed in future work. However, the controller did produce healthy-like gait in paraplegic subjects of different anthropometry and walking abilities and at multiple speeds, thus demonstrating high robustness. These additional features may, therefore, not be necessary.

We conducted this study on a treadmill but aim to demonstrate that the NMC can also provide faster walking speeds overground, beyond the speeds currently reported (0.26 m/s on average, Louie et al., 2015). Using the same controller on a wearable exoskeleton overground poses new challenges, especially for subjects with inadequate volitional hip control. Indeed the treadmill moves the subjects' feet, which could aid in initiating or sustaining gait. The NMC is also better suited for walking at normal to fast speeds and therefore may need to be combined with new algorithms, likely with pre-determined gait patterns. These preset algorithms would contribute more during transient behaviors (e.g., gait initiation and termination) and slow walking speeds. At faster speeds (around 0.6 m/s and greater), the NMC would then fully take control.

## 5. Conclusion

The bio-inspired NMC controller demonstrated remarkable versatility in generating gait patterns tuned to the subjects' dynamics and producing near-physiological gait at near-normative speeds. The positive SCI subject-machine interaction stemmed from replacing the subject's impaired function with dynamical virtual muscles that require few sensors to generate gait. The power law also enabled indirect evaluation of energetic economy for controllers tested on subjects with impaired gait. These preliminary but auspicious results have important implications toward the exploitation of natural walking dynamics through understanding human biological behavior in the design of controllers for wearable devices that are amenable to various environmental conditions and promote intuitive and unobtrusive human-machine interaction.

## Ethics statement

This study was carried out in accordance with the recommendations of the Medical Research Involving Human Subjects Act (WMO), Medisch Ethische ToetsingsCommissie (METC) Twente with written informed consent from all subjects. All subjects gave written informed consent in accordance with the Declaration of Helsinki. The protocol was approved by the Medisch Ethische ToetsingsCommissie (METC) Twente.

## Author contributions

AW, FD, and EV designed the study and performed the experiment with FT, NT, and HV. AW and TB analyzed and interpreted the data. AW wrote the manuscript, and FD, TB, FT, NT, and EV participated in the design and drafting of the manuscript. EV, HV, and AI were involved in critical revision of the manuscript. All authors read and approved the final manuscript.

### Conflict of interest statement

The authors declare that the research was conducted in the absence of any commercial or financial relationships that could be construed as a potential conflict of interest.
